# Cardiac and noncardiac biomarkers in patients undergoing anthracycline chemotherapy – a prospective analysis

**DOI:** 10.1186/s40959-023-00174-1

**Published:** 2023-04-27

**Authors:** Matthew Dean, Min Jung Kim, Sharon Dimauro, Susan Tannenbaum, Garth Graham, Bruce T. Liang, Agnes S. Kim

**Affiliations:** 1 Department of Medicine, Calhoun Cardiology Center, University of Connecticut School of Medicine, 263 Farmington Avenue, Farmington, CT 06030 USA; 2 Virginia Commonwealth University Health System Internal Medicine Residency, 1101 E. Marshall St, Richmond, VA 23298 USA; 3 Pat and Jim Calhoun Cardiology Center, UConn Health, 300 UConn Health Boulevard, Farmington, CT USA; 4 Carole & Ray Neag Comprehensive Cancer Center, UConn Health, 263 Farmington Avenue, Farmington, CT 06030 USA

**Keywords:** Anthracycline, Chemotherapy, Cardiotoxicity, Biomarker, Troponin, Growth/differentiation factor-15, S oluble suppression of tumorigenesis-2, Myeloperoxidase, Caspase, Ejection fraction

## Abstract

**Background:**

Biomarkers represent a potential tool to identify individuals at risk for anthracycline-induced cardiotoxicity (AICT) prior to symptom onset or left ventricular dysfunction.

**Methods:**

This study examined the levels of cardiac and noncardiac biomarkers before, after the last dose of, and 3–6 months after completion of doxorubicin chemotherapy. Cardiac biomarkers included 5th generation high-sensitivity cardiac troponin T (cTnT), N-terminal pro-brain natriuretic peptide, growth/differentiation factor-15 (GDF-15), and soluble suppression of tumorigenesis-2 (sST2). Noncardiac biomarkers included activated caspase-1 (CASP-1), activated caspase-3, C-reactive protein, tumor necrosis factor-α, myeloperoxidase (MPO), galectin-3, and 8-hydroxy-2’-deoxyguanosine. Echocardiographic data (LVEF and LVGLS) were obtained at pre- and post-chemotherapy. Subanalysis examined interval changes in biomarkers among high (cumulative doxorubicin dose ≥ 250 mg/m^2^) and low exposure groups.

**Results:**

The cardiac biomarkers cTnT, GDF-15, and sST2 and the noncardiac biomarkers CASP-1 and MPO demonstrated significant changes over time. cTnT and GDF-15 levels increased after anthracycline exposure, while CASP-1 and MPO decreased significantly. Subanalysis by cumulative dose did not demonstrate a larger increase in any biomarker in the high-dose group.

**Conclusions:**

The results identify biomarkers with significant interval changes in response to anthracycline therapy. Further research is needed to understand the clinical utility of these novel biomarkers.

**Supplementary Information:**

The online version contains supplementary material available at 10.1186/s40959-023-00174-1.

## Background

Anthracycline-based chemotherapy is a common and effective treatment for various solid and hematologic malignancies, including breast, gynecologic, and bladder cancers, as well as leukemia and lymphoma [1, 2]. Although effective, the use of anthracyclines is limited by its well-established link to cardiotoxicity [2, 3]. The antineoplastic mechanisms of anthracyclines include interaction with topoisomerase-II, DNA intercalation, generation of reactive oxygen species, and systemic inflammation [1, 4–6]. While the exact mechanisms of cardiotoxicity have not been fully elucidated, the double-stranded DNA breaks triggered by the formation of doxorubicin-topoisomerase IIβ complexes meant to kill cancer cells also induce myocyte death [3]. Additionally, iron dysregulation may play a role in anthracycline-induced cardiotoxicity (AICT) as supported by mitochondrial iron accumulation in cardiomyocytes exposed to doxorubicin and the beneficial effects of iron chelation therapy in mitigating cardiotoxicity [7–10]. Patients at increased risk of AICT are those who have received high-dose anthracyclines (cumulative doxorubicin ≥ 250 mg/m^2^ or epirubicin ≥ 600 mg/m^2^) [11, 12]. Additional factors that increase the risk of AICT include history of underlying cardiovascular disease, hypertension, diabetes, obesity, genetic susceptibility, individual differences in drug metabolism, and concomitant exposure to trastuzumab and/or radiation [13–15].

Currently, there is no universal definition for AICT. Diagnosis is made due to new-onset heart failure (HF) or imaging evidence of left ventricular (LV) dysfunction, which is commonly characterized by ≥ 10% decrease in LV ejection fraction (LVEF) to a value less than the lower limit of normal [1, 16]. A relative reduction of > 15% in LV global longitudinal strain (LVGLS) is a useful parameter to predict AICT [17, 18]. Impairment in myocardial deformation precedes contractile dysfunction. Studies using various definitions of AICT estimate the incidence rate to be 2.2-9% [11, 19, 20]. The current diagnostic approach lacks the sensitivity to detect early subclinical cardiac dysfunction and cannot reliably predict future outcomes [21, 22]. An improved predictive model would be able to detect AICT prior to decreases in LVEF and symptom onset and would include both LVGLS and biomarkers (BM). Both cardiac and noncardiac BMs have been investigated as potential indicators of cardiotoxicity which could aid in the early identification and diagnosis of AICT.

Cardiac BMs that have received the most attention include troponin and natriuretic peptides [23, 24]. Troponin has been the most well-studied BM to date. Analyses have identified troponin levels to increase with anthracycline therapy and found associations between troponin levels and changes in LVEF [25, 26]. Studies examining natriuretic peptides, both brain natriuretic peptide (BNP) and N-terminal pro-brain natriuretic peptide (NT-proBNP), have been inconclusive with contradicting results [27–31]. Additional cardiac BMs studied include growth/differentiation factor-15 (GDF-15) and soluble suppression of tumorigenesis-2 (sST2), however their role is currently undetermined [32–34].

Without conclusive evidence to establish a cardiac BM screening protocol, noncardiac BMs have also garnered attention. Limited data are currently available to assess the relationship of noncardiac BMs to AICT, however those that have been examined include C-reactive protein (CRP), myeloperoxidase (MPO), placental growth factor (PlGF), soluble fms-like tyrosine kinase receptor (sFlt)-1, and galectin-3 (Gal3) [22, 23, 33, 35].

The main objective of this study was to examine the changes in the levels of cardiac and noncardiac BMs in individuals exposed to doxorubicin at three time points: before, after the last dose of, and 3–6 months after completion of chemotherapy. Echocardiographic data were obtained to follow changes in LVEF and LVGLS with anthracycline exposure. Interval changes in BM levels were compared between individuals who received high cumulative doxorubicin dose (≥ 250 mg/m^2^) vs. low dose (< 250 mg/m^2^).

BMs were selected based upon their physiologic relevance to AICT and their potential for early cardiotoxicity detection. Troponins, which reflect cardiomyocyte injury and necrosis, may allow the detection and quantification of myocyte death after exposure to anthracycline [36, 37]. Interestingly, persistent troponin elevation after doxorubicin exposure may signify ongoing injury and predict AICT [26]. NT-proBNP, a neurohormone released by the cardiomyocytes in response to cardiac wall stress, may reflect hemodynamic and structural changes within the myocardium to indicate myocardial dysfunction following anthracycline therapy. Higher levels of natriuretic peptides have been identified in those with asymptomatic cardiac dysfunction suggesting they may be elevated prior to symptom onset, facilitating earlier diagnosis of AICT [38, 39]. They may also serve as a prognostic marker for those who have established LV dysfunction [40]. Furthermore, oxidative stress, DNA damage, and systemic inflammation are integral mechanisms of AICT. Thus, markers of inflammation CRP, caspase-1 (CASP-1) and tumor necrosis factor-α (TNF-α), as well as markers of oxidative stress 8-hydroxy-2’-deoxyguanosine (OHDG), MPO, and GDF-15 were evaluated in this study. Caspase-3 (CASP-3), which executes programmed cell death and is a marker of apoptosis, was hypothesized to be implicated in AICT. In addition, CASP proteins have been found to be elevated in patients with HF [41, 42], suggesting they may be useful markers of chronic stages of AICT. BMs of myocardial fibrosis, a downstream effect of ventricular remodeling, are likely elevated in later stages of AICT. In mouse models, cardiac magnetic resonance imaging was able to detect evidence of fibrosis starting at four weeks of anthracycline administration [43]. BMs of fibrosis, such as Gal3 and sST2, were evaluated in this study.

## Methods

### Study procedure

Recruitment of study patients occurred prospectively at the University of Connecticut Health Center from January 2016 to March 2020. The target population comprised of male and female subjects over the age of 18 who were diagnosed with hematologic or solid tumor malignancies and who were scheduled to receive a doxorubicin-containing chemotherapeutic regimen. Exclusion criteria included patients (1) who were unable to provide informed consent, (2) who had undergone cardiac or vascular surgery, percutaneous coronary angioplasty (PCI), or pacemaker/internal defibrillator placement within the 3 months prior to enrollment, (3) who had myocardial infarction within the 3 months prior to enrollment, (4) who were pregnant, and (5) who were being treated for cancer recurrence.

Serum BMs were obtained at three time points: (1) Pre-Chemo (prior to the initiation of chemotherapy), (2) End-Chemo (after the last dose of anthracycline), and (3) Post-Chemo (3 to 6 months after completion of anthracycline). A sample of blood (10 mls: 3 gold tops and 1 purple top) was taken to measure the levels of circulating BMs. Eleven BMs were tested, and these BMs consisted of cardiac BMs: 5th generation high sensitivity cardiac troponin (cTnT), NT-proBNP, GDF-15, and sST2 and noncardiac BMs: CASP-1, CASP-3, CRP, TNF-α, Gal3, OHDG, and MPO. Normal values for these BMs can be found in Supplemental Table 1 [42].

The echocardiographic imaging parameters were collected at two time points: (1) Pre-Chemo (prior to the initiation of chemotherapy) and (2) Post-Chemo (3 to 6 months after completion of anthracycline chemotherapy). Patients underwent transthoracic echocardiogram with the Philips IE33 system (Philips Medical Systems, Andover, MA, USA) for the assessment of LV cavity size, LVEF, and LVGLS. Standardized transthoracic echocardiographic examinations were performed under continuous electrocardiographic recording. Data acquisition was completed by a 5–1 MHz sector transducer in the parasternal and apical views. The biplane method of disks (modified Simpson’s method) according to the American Society of Echocardiography guidelines was used for the determination of LVEF. Longitudinal end-systolic strain was measured at the endocardial border using speckle-tracking echocardiography. LVGLS was calculated by averaging the peak longitudinal strain values of three apical images (standard 2-, 3- and 4-chamber views) using commercially available software QLAB 9 (cardiac motion quantification; Philips Medical Systems).

Chart review of the enrolled patients was performed to obtain age, gender, ethnicity, race, comorbidity, cancer types, cumulative dose (CD) of anthracycline, and hospitalization outcomes.

### Statistical analysis

All the BM data were visually inspected and tested for normality using the Anderson-Darling test to determine appropriate statistical approaches. All the variables were presented as median with interquartile range (IQR) or percentage if the data did not satisfy normality assumption. Otherwise, descriptive statistics for variables are presented as means with standard deviations. Comparisons of BM levels over time (Pre-Chemo, End-Chemo, and Post-Chemo) were accomplished with the use of Friedman tests followed by post hoc analyses. The Wilcoxon matched pair signed rank test compared BM levels between (1) Pre-Chemo and End-Chemo, (2) Pre-Chemo and Post-Chemo, and (3) End-Chemo and Post-Chemo. Forest plots were used to highlight the fold changes in BMs from Pre-Chemo to End-Chemo and from Pre-Chemo to Post-Chemo time points. In addition, we determined the proportion of cases in which BM levels exceeded the normal range among those BMs that increased from Pre-Chemo to End-Chemo or from Pre-Chemo to Post-Chemo.

To make a paired comparison in echocardiographic data (LVEF and LVGLS), a paired t-test was performed between Pre-Chemo and Post-Chemo. Spearman correlation analysis was performed to analyze the relationship between BM changes (from Pre-Chemo to End-Chemo) and changes in LVEF (from Pre-Chemo to Post-Chemo) and LVGLS (from Pre-Chemo to Post-Chemo).

Cumulative dose (CD) of anthracycline exposure was defined as high (≥ 250 mg/m^2^ doxorubicin) and low (< 250 mg/m^2^ doxorubicin) in accordance with previous literature demonstrating increased prevalence of AICT among those with CD ≥ 250 mg/m^2^. [12, 44]. Fisher’s exact test assessed the relationship between CD and AICT. Once stratified into high and low dose groups, BM interval changes (from Pre-Chemo to End-Chemo) were assessed using Wilcoxon signed rank test. BM interval changes were then compared among the subgroups.

## Results

### Participant characteristics

Table 1 presents the baseline demographic and clinical characteristics of the study population. Participants enrolled in this study were mostly younger adults (70%) < 65 years old, female (61%), Caucasian (52%), and had breast cancer (46%).

**Table 1:** Baseline characteristics of the study patient cohort

Variable	Values (n = 41)
Age (average ± Std)	55.73 ± 15.72
RACE	
White	34 (83%)
Black	3 (7%)
Hispanic	4 (10%)
Female	25 (61%)
Comorbidity	
Hypertension	11 (27%)
Diabetes Mellitus	4 (10%)
CAD	3 (7%)
CKD	4 (10%)
Cancer Type	
Breast	19 (46%)
Lymphoma	14 (34%)
Others	8 (20%)

### Cardiac biomarkers

**Fig. 1:**
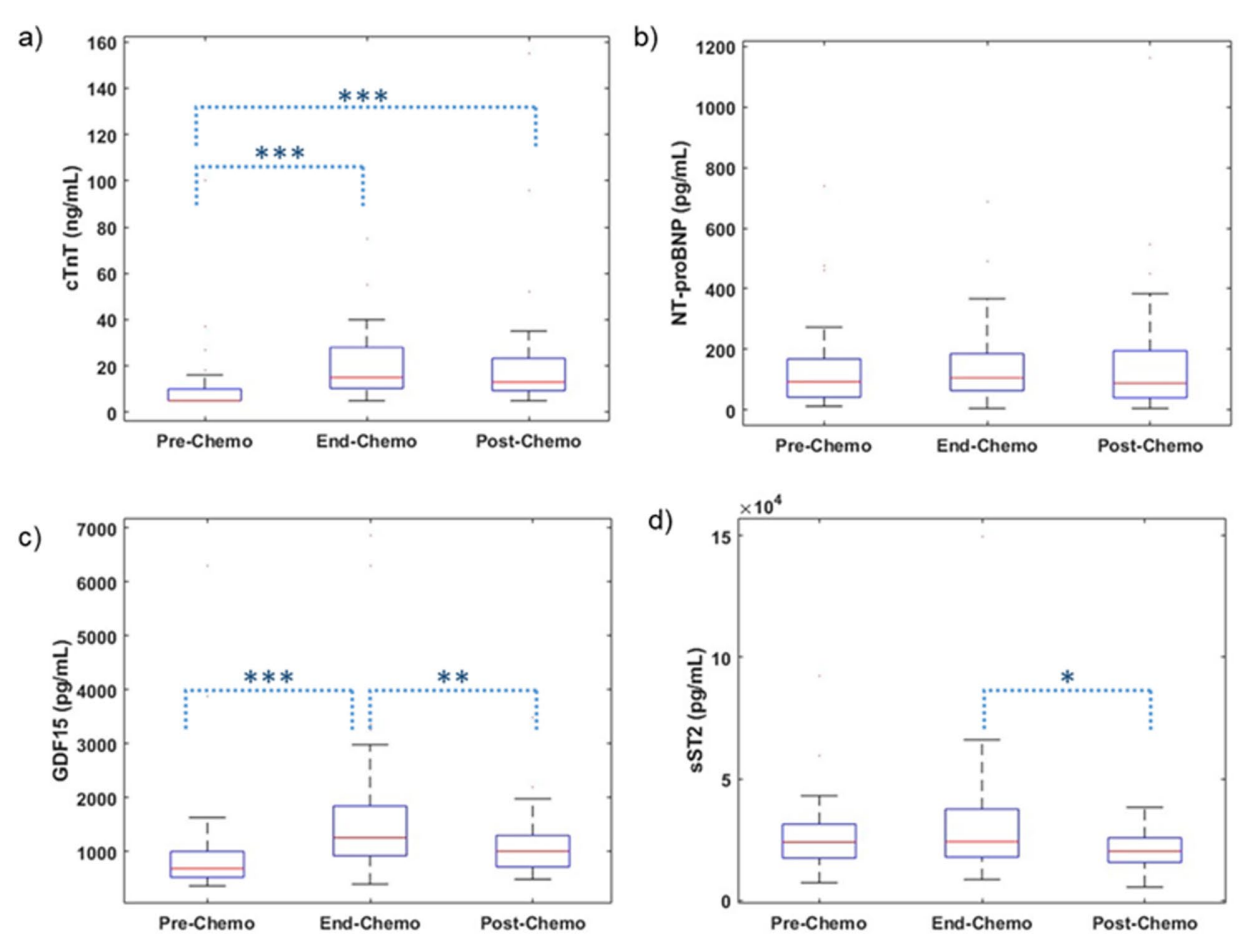
Changes in cardiac biomarker levels at Pre-Chemo, End-Chemo, and Post-Chemo. (**a**) 5th generation high-sensitivity cardiac troponin T (cTnT), (**b**) N-terminal pro-brain natriuretic peptide (NT-proBNP), (**c**) growth/differentiation factor-15 (GDF-15), and (**d**) soluble suppression of tumorigenesis-2 (sST2) biomarker levels at three time points: prior to chemotherapy (Pre-Chemo), after the last dose of doxorubicin (End-Chemo), and 3-6 months after completion of doxorubicin (Post-Chemo). *** p < 0.001, ** p  < 0.01, * p < 0.05

We sought to see if the levels of cardiac BMs (cTnT, pro-BNP, GDF15, and sST2) changed after chemotherapy (Fig. 1). Cardiac BMs that demonstrated significant differences between time points included cTnT (p < 0.001), GDF-15 (p < 0.001), and sST2 (p = 0.049). cTnT levels increased after chemotherapy: median (IQR) cTnT levels (ng/mL) across Pre-Chemo, End-Chemo, and Post-Chemo were 4.90 (4.90–10.00), 14.00 (10.25–28.00), and 13.00 (9.25–23.25), respectively. Values at End-Chemo (p < 0.001) and Post-Chemo (p < 0.001) were significantly elevated compared with Pre-Chemo. GDF-15 levels (pg/mL) were the highest at End-Chemo with a median (IQR) of 1259.40 (923.70–1848.08). Median (IQR) GDF-15 levels were 689.70 (525.38–1003.50) at Pre-Chemo and 1006.60 (720.08–1302.00) at Post-Chemo. GDF-15 levels at End-Chemo were significantly increased compared to Pre-Chemo (p < 0.001). A significant decrease in GDF-15 levels was noted from End-Chemo to Post-Chemo (p = 0.007). The median (IQR) of sST2 (pg/mL) at Pre-Chemo, End-Chemo, and Post-Chemo was 23,962.00 (17438.50–31443.25), 24,207.00 (17682.25–37578.25), and 20,207.00 (15702.25–25774.50). A non-significant increase in sST2 levels was noted from Pre-Chemo to End-Chemo (p = 0.054), followed by a significant decrease from End-Chemo to Post-Chemo (p = 0.049).

Figure 3 highlights the increase in BM levels from Pre-Chemo to End-Chemo in cTnT (p < 0.001), GDF-15 (p < 0.001), and sST2 (p = 0.054). The BMs which significantly increased from Pre-Chemo to Post-Chemo were cTnT (p < 0.001) and GDF-15 (p < 0.001). 64% of those patients who had an increase in cTnT from Pre-Chemo to End-Chemo and 61% of those individuals with an increase in GDF-15 had readings above the normal reference range.

**Fig. 2:**
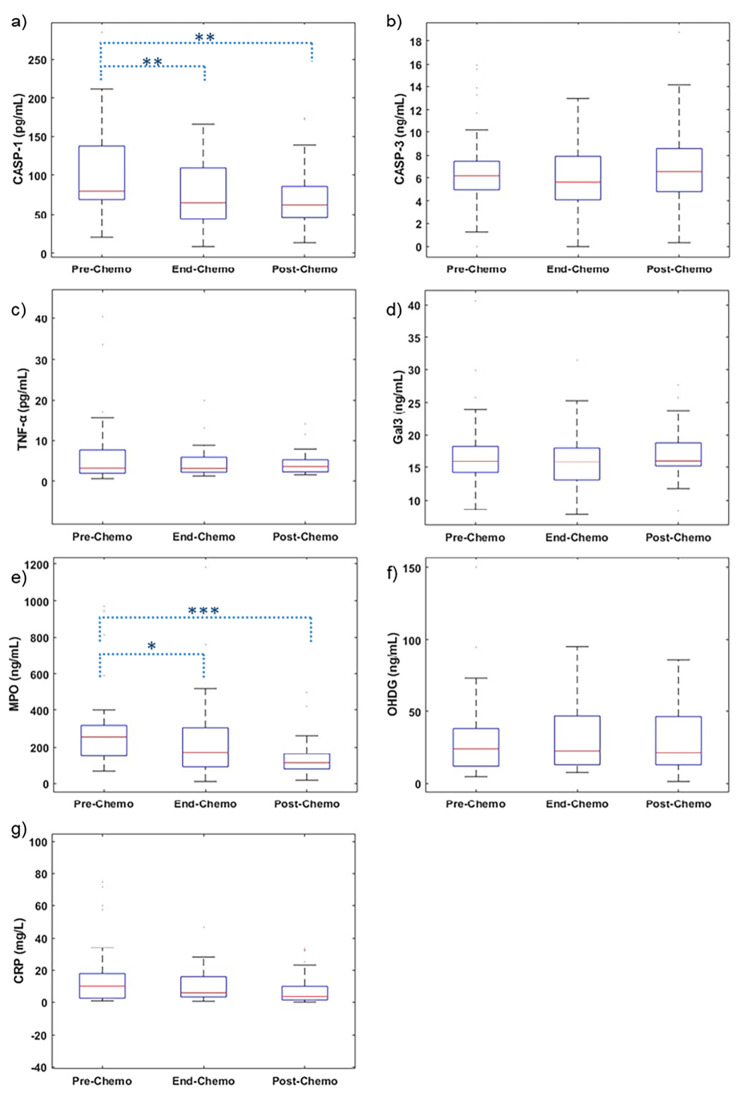
Changes in noncardiac biomarker levels at Pre-Chemo, End-Chemo, and Post-Chemo. (**a**) activated caspase-1 (CASP-1), (**b**) activated caspase-3 (CASP-3), (**c**) tumor necrosis factor-α (TNF-α), (**d**) galectin-3 (Gal3), (**e**) myeloperoxidase (MPO), (**f**) 8-hydroxy-2'-deoxyguanosine (OHDG), (**g**) C-reactive protein (CRP) biomarker levels at three time points: prior to chemotherapy (Pre-Chemo), after the last dose of doxorubicin (End-Chemo), and 3-6 months after completion of doxorubicin (Post-Chemo). *** p < 0.001, ** p < 0.01, * p < 0.05

**Fig. 3:**
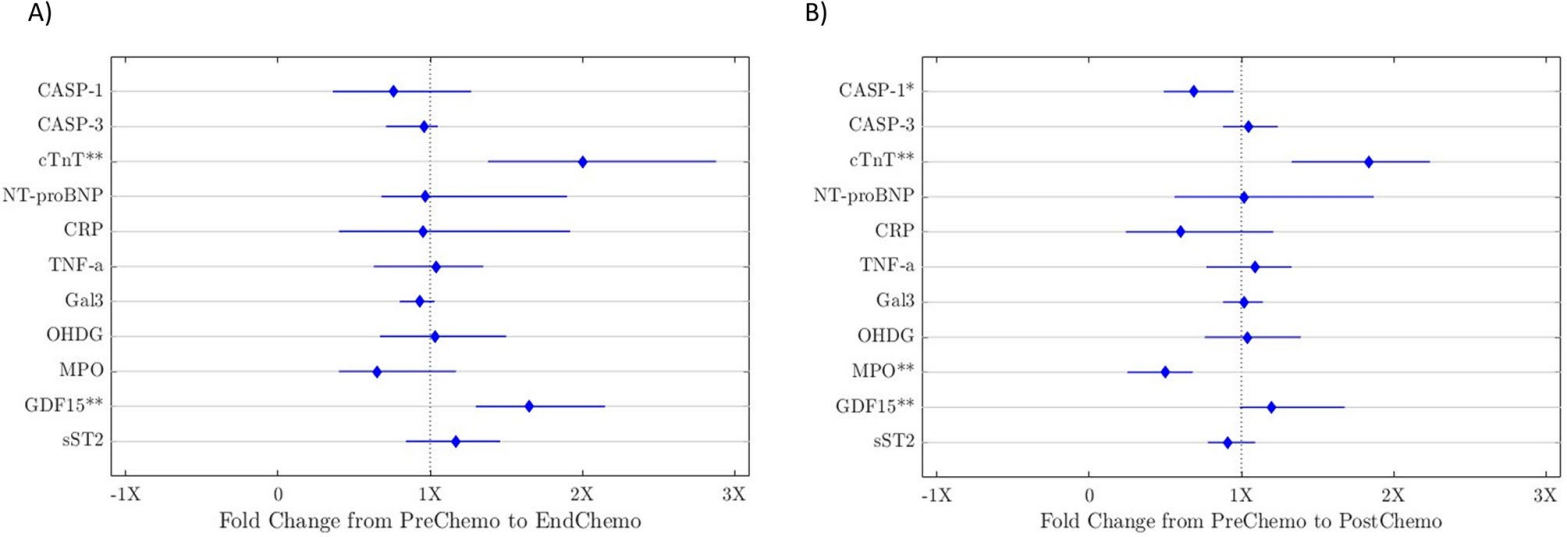
Fold change (increase or decrease) in biomarker levels (**A**) from Pre-Chemo to End-Chemo, (**B**) from Pre-Chemo to Post-Chemo. The horizontal line represents the median and interquartile ranges of the fold changes in biomarkers. **p < 0.001; *0.01 ≤ p < 0.05

### Noncardiac biomarkers

To determine whether noncardiac BM levels changed after chemotherapy, the levels of CASP-1, CASP-3, CRP, TNF-α, Gal3, OHDG, MPO were measured across Pre-Chemo, End-Chemo, and Post-Chemo (Fig. 2). Noncardiac BMs that demonstrated significant differences between time points included CASP-1 (p = 0.002) and MPO (p < 0.001). CASP-1 levels (pg/mL) decreased at each time point. The median (IQR) CASP-1 levels across Pre-Chemo, End-Chemo, and Post-Chemo were 79.56 (68.94–137.90), 65.10 (43.43–110.31), and 62.08 (46.31–85.63), respectively. Significant decreases in CASP-1 levels were noted from Pre-Chemo to End-Chemo (p = 0.008) and from Pre-Chemo to Post-Chemo (p = 0.006). MPO also showed a decreasing pattern with highest level at Pre-Chemo. The median (IQR) of MPO levels (ng/mL) was 255.00 (150 − 13–317.58) at Pre-Chemo, 171.00 (90.48–304.78) at End-Chemo, and 112.10 (78.80–162.03) at Post-Chemo. MPO was significantly higher at Pre-Chemo than at End-Chemo (p = 0.049) and at Post-Chemo (p < 0.001). As seen in Fig. 3, we observed a significant decrease in CASP-1 (p = 0.048) and MPO (p < 0.001) from Pre-Chemo to Post-Chemo.

### Relation to echocardiographic parameters

LVEF significantly decreased from baseline to Post-Chemo (Mean ± STD: 63.14 ± 5.47% vs. 57.3 ± 6.48%; p < 0.0001), and LVGLS also significantly decreased from baseline to Post-Chemo (Mean ± STD: -19.50 ± 3.18% vs. -18.56 ± 3.22%; p = 0.04). To ascertain the relationship between the BM interval changes (from Pre-Chemo to End-Chemo) and differences in echocardiographic parameters (LVEF and LVGLS) from Pre-Chemo to Post-Chemo, Spearman correlations were performed. BMs found to correlate with LVEF changes were cTnT (ρ = 0.325, p = 0.046), and Gal3 (ρ = 0.407, p = 0.011). No BMs were found to correlate with LVGLS.

### High-dose vs. low-dose anthracycline

Out of 41 patients, 14 (34.1%) patients were defined as having received high CD, whereas 27 were found to be in the low CD group. Table 2 presents the baseline patient characteristics of the high- and low-dose groups. The distribution of cancer type was significantly different between the two groups, with a higher proportion of patients having lymphoma and a lower proportion having breast cancer in the high-dose group compared to the low-dose group. Among the high-dose group, BMs with significant interval change included CASP-1 (p = 0.04), CASP-3 (p = 0.02), MPO (p = 0.04), cTnT (p = 0.02), and GDF-15 (p < 0.0001). Median [IQR] BM changes (End-Chemo - Pre-Chemo) were − 32.70 [-57.81 - -12.17] in CASP-1, -0.90 [-2.14–0.05] in CASP-3, -75.40 [-214.60–5.70] in MPO, 4.10 [0.00–19.00] in cTnT, and 326.30 [97.10–708.20] in GDF-15. Among the low-dose group, BMs with significant interval change included cTnT (p < 0.0001) and GDF-15 (p < 0.0001). The median [IQR] of BM changes (End-Chemo - Pre-Chemo) were 8.10 [3.03–12.58] in cTnT, and 495.00 [208.93–875.40] in GDF-15. No significant difference in BM change was noted when the high- and low-dose groups were directly compared.

**Table 2:** Baseline characteristics among high- and low-dose groups

Variable	High-Dose (n = 14)	Low-Dose (n = 27)		p_Value	
Age (average ± Std)	60.46 ± 15.80	53.44 ± 15.44		0.19	
RACE				0.447	
White	11 (79%)	23 (85%)			
Black	2 (14%)	1 (4%)			
Hispanic	1 (7%)	3 (11%)			
Female	6 (43%)	19 (70%)		0.087	
Comorbidity					
Hypertension	6 (43%)	5 (19%)		0.217	
Diabetes Mellitus	3 (21%)	1 (4%)		0.07	
CAD	2 (14%)	1 (4%)		0.217	
CKD	2 (14%)	2 (7%)		0.482	
Cancer Type				< 0.001	
Breast	0 (0%)	19 (70%)			
Lymphoma	12 (86%)	2 (7%)			
Others	2 (14%)	6 (22%)			

## Discussion

In total, 5 BMs (3 cardiac and 2 noncardiac) demonstrated significant differences between time intervals. All BMs were hypothesized to increase with exposure to anthracyclines based on the postulated mechanisms of AICT, which include inflammation, oxidative stress, myocyte injury, and cardiac wall stress. cTnT and GDF-15 had significant increases from Pre-Chemo to End-Chemo time points, as expected. sST2 also had a non-significant increase from Pre-Chemo to End-Chemo. cTnT was also significantly higher at Post-Chemo compared to Pre-Chemo, suggesting potential ongoing myocardial injury after completion of therapy. However, a non-significant decrease in cTnT occurred from End-Chemo to Post-Chemo, suggesting that the myocardial injury process slows down after the completion of chemotherapy. Interestingly, GDF-15 was found to significantly decrease from End-Chemo to Post-Chemo, corresponding with the withdrawal of chemotherapy. GDF-15, a cytokine belonging to the transforming growth factor family, is overexpressed in stress and inflammatory conditions, such as myocardial ischemia, cardiac pressure overload conditions, and cancer [45]. As a marker of inflammation and oxidative stress, GDF-15 has been associated with adverse prognosis in cardiovascular disease, including coronary artery disease, atrial fibrillation and heart failure [46]. In various cancers, higher GDF-15 levels were associated with worse disease-free and overall survival [45]. In our study, GDF-15 increased from baseline to End-Chemo, which could be a marker of cardiotoxicity. It then decreased from end of chemotherapy to 3–6 months post chemotherapy, potentially indicating effective cancer treatment.

MPO and CASP-1 significantly decreased from Pre-Chemo to End-Chemo and from Pre-Chemo to Post-Chemo. This finding is the opposite of what was hypothesized given the role of MPO in oxidative stress and the relationship of CASP-1 protein to inflammation. The observed decreases in these BMs with anthracycline exposure suggest that some patients with cancer have elevated MPO and CASP-1 levels at baseline because of the systemic inflammation related to cancer. Thus, antineoplastic treatment resulted in decreased, rather than elevated, MPO and CASP-1 levels. MPO has been proposed as a tumor marker in hematologic malignancies and has been demonstrated to be elevated at baseline prior to treatment with anthracyclines [22, 47]. The results suggest that following these levels may be useful in monitoring the oncologic response to cancer treatment.

When classified according to CD of doxorubicin, interval changes in BM levels were not significantly different between the high- and low-dose groups. cTnT and GDF-15 were the only BMs with a significant increase from Pre-Chemo to End-chemo and from Pre-Chemo to Post-Chemo, an observation that was seen in both CD groups. This finding is contrary to the expected association of AICT with anthracycline dose. Cumulative doxorubicin dose ≥ 250 mg/m^2^ has been associated with a greater incidence of cardiotoxicity. Our finding suggests that there is no safe dose of anthracyclines, and that other factors, such as genetic predisposition and individual differences in drug metabolism, contribute to the development of AICT. In addition, the risk factors for AICT include preexisting cardiac conditions, hypertension, DM, and obesity [14]. The baseline characteristics between the high- and low-dose groups are largely similar except for the type of malignancy. Patients with lymphoma are more likely to receive a high-dose regimen. Interestingly, CASP-1, CASP-3 and MPO had significant decreases from Pre-Chemo to End-Chemo in the high-dose cohort but not in the low-dose group. Whether these BMs reflect effective cancer regression with treatment remains to be studied in future studies.

**Fig. 4:**
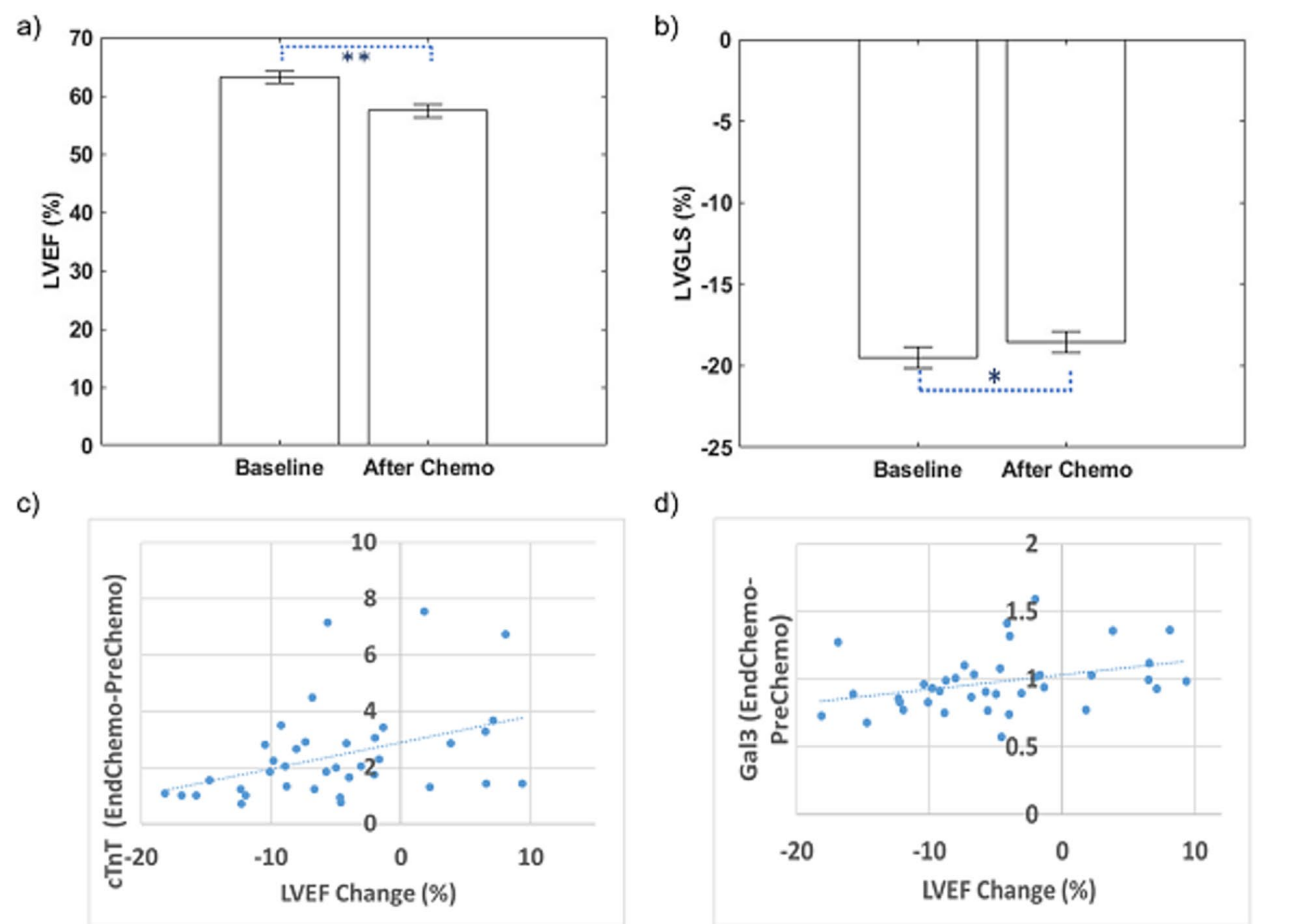
Analysis of (**a**) left ventricular ejection fraction (LVEF) and (**b**) left ventricular global longitudinal strain (LVGLS) with anthracycline exposure. (**c**) Correlation of change in LVEF with interval change in 5th generation high-sensitivity cardiac troponin T (cTnT) from Pre-chemo to End-Chemo. (**d**) Correlation of change in LVEF with interval change in galectin-3 (Gal3) from Pre-Chemo to End-Chemo. ** p < 0.0001, * p < 0.05

Except for Gal3 and cTnT, none of the BMs correlated with changes in LVEF or LVGLS. A positive correlation was identified between changes in LVEF and cTnT levels, suggesting that those with higher cTnT values also had an increase in LVEF. However, Fig. 4c shows that most patients with an increase in cTnT also had reduction in LVEF, and the positive correlation was driven by two outliers that had large cTnT increases and an increase in LVEF.

These results identify BMs with significant interval changes over time. The markers which increased with anthracycline exposure included cTnT, GDF-15, and sST2. They represent novel BMs with potential ability to predict AICT. Extensive data are currently available on the role of troponin I in AICT early detection and monitoring [31, 48, 49]. Much less data exist on cTnT, and our results support existing studies which demonstrate significant increases in troponin in individuals receiving anthracycline [50, 51]. The finding of significant increase in GDF-15 with anthracycline exposure supports the existing literature [34], while studies to date examining sST2 have yielded mixed results [24, 33].

In this study, NT-proBNP, CRP, TNF-α, CASP-3, OHDG, and Gal3 did not demonstrate significant changes over time. Given the uncertainty in previous research regarding the role of BNP as a BM for AICT, our results help provide clarity demonstrating that its levels do not increase with exposure. The results also expand the current body of evidence on noncardiac BMs, suggesting that noncardiac BMs may not be optimal tools for AICT screening as none of them had significant increases with doxorubicin exposure.

Limitations of the study include a limited sample size. Since patients with cancer undergo frequent blood draws and imaging studies for clinical reasons, such as assessing response to cancer therapy and screening for toxicities, some patients felt over-burdened with having additional bloodwork and cardiac imaging studies for research purposes. Additionally, many patients enrolled in late 2019 and early 2020 did not follow through with serial bloodwork and echocardiograms due to the COVID-19 pandemic and thus had to be excluded. A separate limitation included the fact that echocardiographic data were not obtained at three time points. The largest increases in BMs were seen at the EndChemo period; however, there was no scheduled echocardiogram at this time. This limitation precluded making direct associations between BM increases and changes in LVEF and LVGLS. Lastly, data on the use of cardioprotective medications, such as angiotensin converting enzyme inhibitors/ angiotensin II receptor blockers, and beta-blockers, were not included. In future work, determining whether the use of neurohormonal blockade affects the development of AICT would be valuable.

Further research is needed to clarify the relationship between BM changes and clinical outcomes. In the interim, these results help to advance the understanding of BMs whose levels vary directly with anthracycline exposure. This study sets the stage for future investigation on this important topic.

## Electronic supplementary material

Below is the link to the electronic supplementary material.

Supplementary Material 1

## Data Availability

The data presented in this study are available on request from the corresponding author. The data are not publicly available due to privacy and confidentiality.

## Abbreviations

AICT: anthracycline-induced cardiotoxicity
HF: heart failure
LV: left ventricle
LVEF: left ventricular ejection fraction
BM: biomarkers
BNP: brain natriuretic peptide
NT-proBNP: N-terminal pro-brain natriuretic peptide
GDF-15: growth/differentiation factor-15
sST2: soluble suppression of tumorigenesis-2
CRP: C-reactive protein
MPO: myeloperoxidase
PlGF: placental growth factor
sFlt: oluble fms-like tyrosine kinase receptor
Gal3: galectin-3
TNF-α: tumor necrosis factor-α
SGML: Standard generalized markup language
CASP–: Caspase proteins
OHDG: 8-hydroxy-2’ –deoxyguanosine
PCI: percutaneous coronary angioplasty
cTnT: 5^th^ generation high sensitivity cardiac troponin
CD: cumulative dose
IQR: interquartile range
