# Supplementary material for: Cardiac and noncardiac biomarkers in patients undergoing anthracycline chemotherapy – a prospective analysis
Source: Cardiooncology. 2023 Apr 27;9:23. doi: 10.1186/s40959-023-00174-1 (PMC10133897; doi:10.1186/s40959-023-00174-1)
Supplement: Supplementary file 1 — Supplementary Material 1 [file 40959_2023_174_MOESM1_ESM.docx]

| **Company** | **Biomarker** | **Reference Range** | **Within-Run**  **(Intraassay) CV%** | **Between-Run**  **(Intraassay) CV%** | **Sensitivity** |
| --- | --- | --- | --- | --- | --- |
| Siemens Healthcare Diagnostics, Formerly: Diagnostic Products Corporation | hsCRP | < 3 mg/L | 3.3 | 4.5 | 0.1 mg/L |
| BioVision Incorporated | 8-OHdG | Assay range  1.563 - 100 ng/ml | 9 |  | <0.94 ng/mL |
| R&D Systems, Inc Bio-Techne Brand | GDF-15 | 337-1060 pg/mL | 2.3 | 5.4 | 2.0 pg/mL |
| R&D Systems, Inc Bio-Techne Brand | Myeloperoxidase | 21.4-229 ng/mL | 2.1 | 9 | 0.014 ng/mL |
| ARUP (#2007138) | Galectin 3 | ≤22.1 ng/mL  Low risk: ≤ 17.8ng/mL  Intermediate risk: 17.9-25.9ng/mL  High Risk: > 25.9ng/mL |  |  |  |
| ARUP (#0050083) | proBNP | 0-74 years - ≤ 124pg/mL  ≥75 years - ≤ 449 pg/mL |  |  |  |
| Human Magnetic Cardiac Panel A  (LUCAM523) | ST2/IL-33R | Males 10400-52100 pg/ml  Females 8400-33600 pg/ml |  |  | 6 pg/mL |
| Human High Sensitivity Cytokine Magnetic Panel B (LHSCM210) | TNF-a | ≤22 pg/mL |  |  | 0.54 pg/mL |
| ARUP (#3001831) | Troponin T (cTnT) 5th Generation | Female - ≤10 ng/L  Male - ≤ 15 ng/L |  |  |  |
| R&D Systems Human Caspase-1/ICE Quantikine ELISA kit(DCA100) | Caspase-1 | Assay range  6.25-400pg/mL  Median IQR in healthy subjects:  101.52 [79.97 – 131.94]^42^ |  |  | 1.24pg/mL |
| Cell Signaling Technology PathScan Cleaved Caspase-3 (Asp 175) Sandwich ELISA kit (#7190C) | Caspase-3 | Assay range  0.2-1.3mg/mL  Medial IQR in healthy subjects:  0.998 [0 – 5.44]^42^ |  |  |  |

Supplemental table 1. Normal ranges for biomarkers.
